# Investigating maternal and neonatal health outcomes associated with continuing or ceasing dexamphetamine treatment for women with attention-deficit hyperactivity disorder during pregnancy: a retrospective cohort study

**DOI:** 10.1007/s00737-024-01450-4

**Published:** 2024-03-01

**Authors:** Danielle J. Russell, Caitlin S. Wyrwoll, David B. Preen, Erin Kelty

**Affiliations:** 1 School of Population and Global Health, University of Western Australia, Crawley, WA Australia; 2 School of Human Sciences, University of Western Australia, Crawley, WA Australia

**Keywords:** Attention-deficit hyperactivity disorder (ADHD), Dexamphetamine, Pregnancy, Maternal outcomes, Neonatal outcomes

## Abstract

**Purpose:**

Attention-deficit hyperactivity disorder (ADHD) is becoming more commonly diagnosed in women, consequently, more women of reproductive age are taking ADHD medication, such as dexamphetamine. However, the safety associated with continuing or ceasing dexamphetamine during pregnancy is unclear. This study investigates outcomes associated with the continuation of dexamphetamine during pregnancy compared to those who ceased or were unexposed.

**Methods:**

A population-based retrospective cohort of women from Western Australia who had been dispensed dexamphetamine during pregnancy and gave birth between 2003 and 2018. Women had either continued to take dexamphetamine throughout pregnancy (continuers, *n* = 547) or ceased dexamphetamine before the end of the second trimester (ceasers, *n* = 297). Additionally, a matched (1:1) comparison group of women who were dispensed an ADHD medication prior to pregnancy but not during pregnancy (unexposed) was included in the study (*n* = 844). Multivariable generalised linear models were used to compare maternal and neonatal health outcomes.

**Results:**

Compared to continuers, ceasers had greater odds of threatened abortion (OR: 2.28; 95%CI: 1.00, 5.15; *p* = 0.049). The unexposed had some benefits compared to the continuers, which included lower risk of preeclampsia (OR: 0.58; 95%CI: 0.35, 0.97; *p* = 0.037), hypertension (OR: 0.32; 95%CI: 0.11, 0.93; *p* = 0.036), postpartum haemorrhage (OR: 0.57; 95%CI: 0.41, 0.80; *p* = 0.001), neonatal special care unit admittance (OR: 0.16; 95%CI: 0.12, 0.20; *p* < 0.001) and fetal distress (OR: 0.73; 95%CI: 0.54, 0.99; *p* = 0.042).

**Conclusion:**

Continuing dexamphetamine throughout pregnancy was not associated with an increase in adverse neonatal and maternal health outcomes compared to ceasing. Ceasing dexamphetamine during pregnancy was associated with increased odds of threatened abortion compared with continuing dexamphetamine. However, this is something that requires further investigation due to the small sample size, difficulties examining timing, and the inability to examine spontaneous abortions. The unexposed showed some benefits compared to the continuers, suggesting that where possible the cessation of dexamphetamine prior to pregnancy may be advisable.

## Introduction

Women in their reproductive years are commonly prescribed dexamphetamine (DEX) for the treatment of attention-deficit hyperactivity disorder (ADHD) in Australia (Kittel-Schneider et al. 2021; Oei et al. 2012). The safety of DEX in pregnancy has not been adequately demonstrated, thus it is generally recommended where possible women cease taking DEX when intending to become pregnant (Besag 2014; Kittel-Schneider et al. 2021; Poulton et al. 2018). However, unplanned pregnancies can occur, resulting in unintentional fetal exposure (Koren et al. 2020). The exact prevalence of worldwide psychostimulant medication use in pregnant women with ADHD is not known (Andrade 2018). However, a study conducted in Norway and Sweden established that in 2019, the prevalence of ADHD medication use during pregnancy was 5.5/1000 and 7.9/1000 respectively (Cohen et al. 2023). If women become pregnant while on DEX, physicians typically advise patients to either reduce or cease DEX, to reduce exposure and potential harm to the developing fetus (Besag 2014; Kittel-Schneider et al. 2021; Poulton et al. 2018). However, this advice is based on limited empirical evidence.

Our understanding of the safety of DEX in pregnancy has largely been taken from studies on the use of stimulants more broadly, including studies examining ADHD medication (often with no delineation between medications) (Bro et al. 2015; Hærvig et al. 2014; Källén et al. 2013; Nörby et al. 2017b; Poulton et al. 2018) and studies examining the use of illicit stimulants such as methamphetamine (Gorman et al. 2014; Kalaitzopoulos et al. 2018a; Oei et al. 2010; Thaithumyanon et al. 2005). The use of ADHD medications has been associated with increased risk of being admitted to the neonatal intensive care unit (Nörby et al. 2017a), pre-term birth (Källén et al. 2013; Nörby et al. 2017b; Poulton et al. 2018), low Apgar scores (< 7 or < 10, depending on the study) (Bro et al. 2015; Poulton et al. 2018), and miscarriage (Bro et al. 2015; Hærvig et al. 2014). However, many studies on the safety of ADHD medications in pregnancy are complicated by the contribution of ADHD to adverse maternal and neonatal outcomes (Poulton et al. 2018). Similarly, studies examining the use of illicit stimulants in pregnancy are complicated by confounding factors relating to illicit substance use. Illicit stimulant use during pregnancy has been associated with an increased risk of perinatal mortality (Gorman et al. 2014), pre-term birth (Gorman et al. 2014; Oei et al. 2010), low birth weight (Kalaitzopoulos et al. 2018b), and low Apgar scores (Kalaitzopoulos et al. 2018b). Additionally, neonatal abstinence syndrome has been documented in neonates exposed to illicit amphetamines and methamphetamine (Oei et al. 2010; Thaithumyanon et al. 2005). However, it is difficult to distinguish the direct and indirect effects of illicit stimulants on perinatal health outcomes, due to the high presence of confounding variables.

Given the possibility of harm associated with ADHD medication taken during pregnancy, women may choose to cease their medication during their pregnancy. However, untreated ADHD can be associated with exacerbation of the features of ADHD, causing increased levels of stress, anxiety and depression, which are associated with adverse maternal and neonatal outcomes (Damer et al. 2021; Glover 2013; Grigoriadis et al. 2013). As yet, a direct comparison between outcomes in women who choose to continue or cease ADHD treatment has not been carried out; thus it is difficult to ascertain how the benefits to continue or cease treatment may affect neonatal outcomes (Anderson et al. 2018; Damer et al. 2021). Additionally, little is known about the safety of DEX use during pregnancy for the treatment of ADHD. This study aimed to compare maternal and neonatal health outcomes associated with the cessation or continuation of DEX during pregnancy in women with ADHD. Additionally, the study compared outcomes in women who continued DEX throughout pregnancy to an unexposed comparison group.

## Methods

### Study design

This retrospective cohort study utilised routinely-collected administrative-linked data to investigate the maternal and neonatal health outcomes associated with the use of DEX in pregnancy. The study included women with ADHD, who gave birth in Western Australia (WA) between 2003 and 2018.

### Data sources

The Midwives Notification System (MNS), Monitoring of Drugs of Dependence System (MODDS), Hospital Morbidity Data Collection (HMDC) and the WA Registry of Births, Deaths and Marriages were linked by the WA Data Linkage Branch. Linkage was performed using probabilistic matching with clerical review (Gill et al. 1993; Holman et al. 1999). Data from the MNS contained identifying information for both the mother and child, which aided the linking of maternal dispensing data and data pertaining to neonatal outcomes (i.e. hospital and mortality data)(*Guidelines for midwives notification of case attended*, 2021). The MNS contains information regarding all live born and stillborn neonates who were at least 20 weeks of gestational age or greater than 400 g of birth weight when gestational age was unknown (*Guidelines for midwives notification of case attended*, 2021). Medical termination of pregnancies or pregnancy loss prior to 20 weeks gestation are not included in MNS (*Guidelines for midwives notification of case attended*, 2021).

Eligible women were identified by linking data from the MNS to data from the MODDS. The MODDS contains information on the dispensing of all Schedule 8 medications in WA. In Australia, the Therapeutic Goods Administration has classed DEX as a Schedule 8 medicine, meaning that storing, prescribing, and dispensing of DEX is closely monitored, due to its potential for misuse, abuse, and dependence. The MODDS includes a record of the prescription date, dispensing date, the quantity dispensed and strength. The dispensing date was used with the neonate’s date of birth and estimated length of gestation to determine probable exposure.

### Participants

The study included three groups of women who had been diagnosed with ADHD and had given birth during the study period. Women dispensed DEX during pregnancy were included in the study if they had continued DEX during pregnancy (continuers, *n* = 547) or had been on DEX initially but had ceased DEX prior to the end of the second trimester (from conception to day 179) and who did not resume DEX use during pregnancy (ceasers, *n* = 297). The third group (unexposed) included a matched cohort of women with ADHD who had not used DEX or methylphenidate (MPH) during pregnancy, randomly matched 1:1 to women exposed to DEX during pregnancy by smoking status and parity (*n* = 844). Unfortunately, due to the limited number of individuals available for random matching, exact matching was only permissible using two variables (smoking and parity).

The continuers were dispensed at least one script per trimester for the full pregnancy. Women were also classified as continuers if they gave birth prior to the end of the second trimester and had a dispensed script in trimesters one and two. Ceasers had at least two scripts dispensed, either both in trimester one or in trimester one and two (but not in the third trimester). Dispensing in the second trimester was included, as the woman may not have been aware she was pregnant until this time or she may have waited until the second trimester to cease, so as to avoid pregnancy loss due to possible withdrawal symptoms. The unexposed group had been dispensed at least two scripts of either DEX or MPH 29–1826 days prior to conception (but not during pregnancy). These women were mutually exclusive to the continuers and ceasers.

The study cohort was limited to women aged 18–45 years with singleton pregnancies. For women with more than one eligible pregnancy during the study period, only their first pregnancy was included. In this study, a DEX prescription for this study cohort included dexamphetamine sulfate and/or lisdexamfetamine dimesilate (prodrug to dexamphetamine sulfate). Women who were treated with MPH during pregnancy were excluded.

### Outcomes

Primary maternal outcomes investigated included complications during pregnancy and characteristics of labour and delivery, using data from the MNS (*Guidelines for midwives notification of case attended*, 2021). Pregnancy complications included clinically diagnosed preeclampsia, hypertension, antepartum haemorrhage, premature rupture of membranes, threatened abortion (< 20 weeks gestation) and threatened early labour (< 37weeks) (*Guidelines for midwives notification of case attended*, 2021). Characteristics of labour and delivery included precipitate delivery, postpartum haemorrhage (≥ 500 ml), the onset of labour (i.e. spontaneous, induced or a planned caesarean section/delivery) and caesarean section/delivery (both planned and emergency)(*Guidelines for midwives notification of case attended*, 2021).

Given that ADHD patients are at a greater risk of developing comorbid psychiatric illnesses, hospitalisations for mental illness were examined during the third trimester of pregnancy and in the postpartum period (42 days post birth) (Ebejer et al. 2012; Li et al. 2020). Identification of these hospitalisations was determined using hospital morbidity data, which classified inpatient mental health illnesses using ICD-10-AM codes (F00-F99).

Primary neonatal outcomes of interest were obtained from the MNS, which included estimated gestational length (weeks), preterm birth (< 37 weeks), very preterm birth (< 32 weeks), birth size (i.e. weight, length, and head circumference), Apgar score (both at 1 and 5 min, with a low score being less than 7), neonate length of stay in the hospital (days) and admission to the special care unit (SCU) (Guidelines for midwives notification of case attended, 2021). Neonatal abstinence syndrome (NAS) (P96.1) was ascertained from the HMDC using ICD-10-AM codes.

The MNS and WA Death Register provided data on perinatal mortality, which included stillbirths and those who died within the first 28 days after birth. Fetal distress diagnosis at the time of birth was recorded by the MNS.

Percentages of optimal birth parameters were provided by the WA Data Linkage Branch. They are calculated as per Blair et al. (2005), taking into consideration maternal height along with the neonatal gestational age, sex and birth order (Blair et al. 2005). The percentage of neonates born with a low birth weight (less than 2500 g) was also calculated.

### Statistical methods

Characteristics of the women were descriptively summarised for the three groups, which included maternal age, smoking status, diabetes, previous pregnancies, mental health hospitalisation within 5 years prior to conception and during the first trimester, remoteness, and socioeconomic status. Remoteness and socioeconomic status were calculated by the WA Data Linkage Branch using Spectrum software (Pitney Bowes 2015) to perform geocoding, then allocating socio-economic indexes for areas (SEIFA) and remoteness area (RA) in relation to the applicable census year.

To reduce confounding and account for differences that might exist between patients in each group, the non-exposed comparison was matched to exposed patients. The two groups were matched 1:1 on smoking status and parity. Additionally, comparisons between patient characteristics in each group using univariable generalized linear models were used to identify differences between the groups that were later adjusted when comparing outcomes. Models were adjusted for maternal age, and hospitalization for mental health conditions (5 years prior to conception), as well as the matched variables smoking status during pregnancy and parity (0 or 1 + prior pregnancies). These confounding variables were chosen as they have been associated with maternal and/or neonatal outcomes and are commonly used in pharmaco-epidemiological analysis.

The cessation group and the non-exposed group were compared to the continuation group. Maternal and neonatal health outcomes were investigated using multivariable linear regression (estimated gestation, birth weight), logistic regression (threatened abortion, admission to SCU, fetal distress), ordered logistic regression (Apgar scores), negative binomial regression (parity) and multinomial regression (onset of labour). As the matching was only carried out using two variables and would be considered “loosely matched” unconditional logistic regression was used in fitting with work done by Kuo and colleagues (Kuo et al. 2018). A *p*-value of 0.05 was used to identify statistical significance. Data analysis was completed using Stata/SE 17.0 (StataCorp, College Station, Texas, USA, 2021).

There was a small amount of data missing for remoteness, SEIFA, birth length, head circumference, percentage of optimal birth length, percentage of optimal head circumference, and percentage of optimal birth weight. The percentage of missing data was less than 5.3% for these variables. The portion of missing data did not differ between the groups. Where data was missing, these individuals were excluded from the analysis of that outcome.

## Results

### Demographics

A total of 844 eligible women who were dispensed DEX during pregnancy were included in this study. Of these, 547 women (64.8%) were continuers, while 297 (35.2%) were ceasers. For those who ceased using DEX during pregnancy, this typically took place in the second trimester (*n* = 182, 61.3%). There were 844 women in the matched unexposed group. Women in the unexposed group were younger by an average of 2 years (coeff: -2.05; 95%CI: -2.71, -1.39; *p* < 0.001) and had 1.5-times the odds of being hospitalised with mental health conditions within the five years prior to conception than women in the continued group (OR: 1.58; 95%CI: 1.18, 2.12; *p* = 0.002) (Table 1). Ceasers were not significantly different from the continuers in terms of maternal age, smoking status, diabetes diagnosis, number of previous pregnancies, mental health hospitalisations (both five years prior to conception and in the first trimester), and socioeconomic status (Table 1).

**Table 1:** Characteristics of women who continued treatment compared to those who ceased or were unexposed women

	Continuers	Ceasers	Continuers vs. ceasers		Unexposed	Continuers vs. unexposed	
			Effect size (95%CI)	*p*-value		Effect size (95%CI)	*p*-value
Number of women	547	297	-	-	844	-	-
Maternal age (years), mean ± sd	29.2 ± 5.5	28.6 ± 5.9	coeff: -0.59 (-1.45, 0.27)	0.184	27.2 ± 6.5	coeff: -2.05 (-2.71, -1.39)	**0.000**
Smoking, n (%)	167 (30.5%)	92 (31.0%)	OR: 1.02 (0.75, 1.39)	0.893	259 (30.7%)	OR: 1.01 (0.80, 1.27)	0.951
Diabetes, n (%)	42 (7.7%)	19 (6.4%)	OR: 0.82 (0.47, 1.44)	0.493	53 (6.3%)	OR: 0.81 (0.53, 1.23)	0.313
Previous pregnancies, median (IQR)	1 (0, 2)	1 (0, 2)	coeff: -0.03 (-0.19, 0.13)	0.719	1 (0, 2)	coeff: -0.05 (-0.17, 0.07)	0.410
Mental health hospitalisation							
Previous (5 years prior to conception), n (%)	78 (14.3%)	50 (16.8%)	OR: 1.22 (0.83, 1.79)	0.320	176 (20.8%)	OR: 1.58 (1.18, 2.12)	**0.002**
Trimester 1, n (%)	6 (1.1%)	< 5 (< 1.7%)	-^a^	-^a^	7 (0.8%)	OR: 0.75 (0.25, 2.26)	0.614
SEIFA advantage, n (%)							
1 (most disadvantage)	53 (9.7%)	24 (8.1%)	OR: 0.95 (0.74, 1.22)	0.664	95 (11.3%)	OR: 0.91 (0.75, 1.10)	0.318
2	72 (13.2%)	46 (15.5%)			118 (14.0%)		
3	168 (30.7%)	98 (33.0%)			251 (29.8%)		
4	136 (24.9%)	66 (22.2%)			212 (25.1%)		
5 (most advantage)	118 (21.6%)	63 (21.2%)			167 (19.8%)		
Remoteness, n (%)							
Major city	416 (76.0%)	215 (72.4%)	OR: 1.23 (0.90, 1.69)	0.199	613 (72.7%)	OR: 1.24 (0.97, 1.58)	0.087
Inner regional	96 (17.5%)	54 (18.2%)			137 (16.2%)		
Outer regional	22 (4.0%)	19 (6.4%)			61 (7.2%)		
Remote	5 (0.9%)	< 5 (< 1.7%)			24 (2.8%)		
Very remote	8 (1.5%)	< 5 (< 1.7%)			8 (0.9%)		

IQR = interquartile range, n = number, sd = standard deviation, SEIFA = socio-economic indexes for areas, 95%CI = 95% confidence interval, coeff = coefficient, OR = odds ratio

^a^ Data not reported to maintain anonymity

Bold *p*-value indicates statistically significant results (*p* < 0.05)

### Maternal outcomes

Ceasers were more than twice as likely to have a threatened abortion than those in the continued group (OR: 2.28; 95%CI: 1.00, 5.15; *p* = 0.049) (Table 2). There was no difference between the proportion of unexposed and continuers in terms of threatened abortion (OR: 1.13; 95%CI: 0.53, 2.42; *p* = 0.756) (Table 2). There was no significant difference in preeclampsia between the ceasers and continuers (OR: 0.64; 95%CI: 0.32, 1.26; *p* = 0.199) (Table 2). The unexposed group were less likely to develop preeclampsia compared to continuers (OR: 0.58; 95%CI: 0.35, 0.97; *p* = 0.037) (Table 2). The unexposed had a lower risk of developing hypertension compared to the continuers (OR: 0.32; 95%CI: 0.11, 0.93; *p* = 0.036) (Table 2). Less than five ceasers were diagnosed with hypertension making comparisons unreliable. The unexposed women also had a lower risk of having a post-partum haemorrhage compared to the continuers (OR: 0.57; 95%CI: 0.41, 0.80; *p* = 0.001) (Table 2). However, the risk of antepartum haemorrhage did not differ between the two groups (OR: 0.78; 95%CI: 0.46, 1.33; *p* = 0.363) (Table 2). The portion of patients experiencing postpartum and antepartum haemorrhage was not different in ceasers and continuers.

**Table 2:** Maternal health outcomes of women who continued treatment compared to those who ceased or were unexposed women

	Continuers	Ceasers	Continuers vs. ceasers		Unexposed	Continuers vs. unexposed	
			Effect size (95%CI)^a^	*p*-value		Effect size (95%CI)^a^	*p*-value
Preeclampsia, n (%)	33 (6.0%)	12 (4.0%)	OR: 0.64 (0.32, 1.26)	0.199	31 (3.7%)	OR: 0.58 (0.35, 0.97)	**0.037**
Hypertension, n (%)	11 (2.0%)	< 5 (< 1.7%)	-^b^	-^b^	5 (0.6%)	OR: 0.32 (0.11, 0.93)	**0.036**
Threatened abortion, n (%)	11 (2.0%)	13 (4.4%)	OR: 2.28 (1.00, 5.15)	**0.049**	18 (2.1%)	OR: 1.13 (0.53, 2.42)	0.756
Threatened early labour, n (%)	20 (3.7%)	13 (4.4%)	OR: 1.22 (0.60, 2.50)	0.589	19 (2.2%)	OR: 0.63 (0.33, 1.20)	0.157
Premature rupture of membranes, n (%)	36 (6.6%)	11 (3.7%)	OR: 0.55 (0.28, 1.10)	0.092	39 (4.6%)	OR: 0.71 (0.44, 1.14)	0.163
Antepartum haemorrhage, n (%)	27 (5.0%)	12 (4.0%)	OR: 0.82 (0.41, 1.64)	0.569	32 (3.8%)	OR: 0.78 (0.46, 1.33)	0.363
Postpartum haemorrhage, n (%)	81 (14.8%)	38 (12.8%)	OR: 0.83 (0.55, 1.26)	0.382	79 (9.4%)	OR: 0.57 (0.41, 0.80)	**0.001**
Onset of labour, n (%)							
Spontaneous	260 (47.5%)	133 (44.8%)	-	-	394 (46.7%)	-	-
Induced	175 (32.0%)	103 (34.7%)	coeff: 0.16 (-0.17, 0.48)	0.343	292 (34.6%)	coeff: 0.14 (-0.11, 0.40)	0.269
No labour (planned c-section)	112 (20.5%)	61 (20.5%)	coeff: 0.11 (-0.28, 0.49)	0.580	158 (18.7%)	coeff: 0.09 (-0.28, 0.39)	0.513
C-section (emergency or planned), n (%)	208 (38.0%)	107 (36.0%)	OR: 0.95 (0.70, 1.27)	0.715	274 (32.5%)	OR: 0.87 (0.69,1.10)	0.250
Precipitate delivery, n (%)	54 (9.9%)	27 (9.1%)	OR: 0.89 (0.54, 1.44)	0.630	77 (9.1%)	OR: 0.83 (0.57, 1.21)	0.347
Mental health hospitalisation							
Trimester 3, n (%)	76 (13.9%)	32 (10.8%)	OR: 0.70 (0.44. 1.10)	0.122	108 (12.8%)	OR: 0.84 (0.60, 1.17)	0.301
Postpartum (day 0–42), n (%)	6 (1.1%)	< 5 (< 1.7%)	-^b^	-^b^	8 (0.9%)	OR: 0.93 (0.32, 2.73)	0.891

c-section = caesarean section/delivery, n = number, 95%CI = 95% confidence interval, coeff = coefficient, OR = odds ratio

^a^ Adjusted for smoking status, parity, maternal age and mental health hospitalisation five years prior to conception

^b^ Data not reported to maintain anonymity

Bold *p*-value indicates statistically significant results (*p* < 0.05)

All other maternal health outcomes in those who continued DEX throughout pregnancy did not significantly differ from those who ceased DEX or were unexposed. This included most complications during pregnancy, such as threatened early labour, premature rupture of membranes, and antepartum haemorrhage. There were no differences between most of the characteristics of labour and delivery, which included the onset of labour, caesarean section/delivery, and precipitate delivery. Mental health hospitalisations were also not significantly different in the third trimester and postpartum period.

### Neonatal outcomes

Compared with neonates of the continuers, the average length of gestation in the ceasers’ neonates was 0.33 weeks longer (95%CI: 0.04, 0.61; *p* = 0.024) (Table 3). The neonates of the unexposed also had a slightly longer average estimated length of gestation by 0.30 weeks compared with neonates from the continuers group (95%CI: 0.09, 0.52; *p* = 0.006) (Table 3).

**Table 3:** Health outcomes of neonates exposed to dexamphetamine compared to those with early exposure or non-exposure

	Continuers	Ceasers	Continuers vs. ceasers		Unexposed	Continuers vs. unexposed	
			Effect size (95%CI)^a^	*p*-value		Effect size (95%CI)^a^	*p*-value
Sex (male), n (%)	294 (53.8%)	161 (54.2%)	coeff: -0.02 (-0.30, 0.26)	0.887	437 (51.8%)	coeff: 0.07 (-0.14, 0.29)	0.503
Estimated gestational length (weeks), mean ± sd	38.2 ± 1.9	38.5 ± 2.0	coeff: 0.33 (0.04, 0.61)	**0.024**	38.6 ± 2.1	coeff: 0.30 (0.09, 0.52)	**0.006**
Pre-term birth, n (%)	69 (12.6%)	30 (10.1%)	OR: 0.78 (0.49, 1.23)	0.291	78 (9.2%)	OR: 0.73 (0.51, 1.03)	0.076
Very pre-term birth, n (%)	< 5 (< 0.9%)	6 (2.0%)	-^b^	-^b^	11 (1.3%)	-^b^	-^b^
Birth weight (g), mean ± sd	3302 ± 555	3357 ± 577	coeff: 53.72 (-26.03, 133.46)	0.187	3319 ± 580	coeff: 7.67 (-53.89, 69.23)	0.807
Percentage optimal birth weight	100.1 ± 14.1	100.2 ± 2.6	coeff: -0.02 (-2.01, 1.96)	0.980	99.0 ± 14.3	coeff: -1.27 (-2.80, 0.26)	0.104
Low birth weight (below 2500 g), n (%)	43 (7.9%)	22 (7.4%)	OR: 0.94 (0.55, 1.60)	0.823	60 (7.1%)	OR: 0.92 (0.61, 1.40)	0.706
Length at birth (cm), mean ± sd	49.8 ± 3.2	50.1 ± 3.0	coeff: 0.31 (-0.12, 0.75)	0.157	49.9 ± 3.1	coeff: 0.06 (-0.27,0.40)	0.716
Percentage optimal birth length	100.1 ± 5.0	100.4 ± 4.5	coeff: 0.30 (-0.37, 0.97)	0.385	99.9 ± 4.5	coeff: -0.25 (-0.77, 0.27)	0.342
Head circumference (cm), mean ± sd	34.1 ± 2.0	34.2 ± 2.0	coeff: 0.10 (-0.19, 0.38)	0.511	34.2 ± 2.1	coeff: 0.12 (-0.11, 0.34)	0.308
Percentage optimal head circumference	99.3 ± 4.4	99.3 ± 4.5	coeff:- 0.02 (-0.68, 0.64)	0.947	99.5 ± 4.7	coeff: 0.05 (-0.46, 0.56)	0.857
Apgar score at one min, median (IQR)	9 (8, 9)	9 (8, 9)	coeff: -0.07 (-0.35, 0.21)	0.613	9 (8, 9)	coeff: 0.01 (-0.21, 0.23)	0.932
Apgar score at five min, median (IQR)	9 (9, 9)	9 (9, 9)	coeff: 0.14 (-0.21, 0.48)	0.434	9 (9, 9)	coeff: 0.05 (-0.22,0.31)	0.727
Low Apgar score at five min, n (%)	10 (1.8%)	10 (3.4%)	OR: 1.88 (0.77, 4.58)	0.166	20 (2.4%)	OR: 1.35 (0.62, 2.93)	0.456
Length of stay in hospital (days), median (IQR)	3 (2–5)	3 (2–5)	coeff: 0.00 (-0.11, 0.12)	0.956	3 (2–5)	coeff: 0.01 (-0.08, 0.10)	0.791
Admission to special care unit, n (%)	304 (55.7%)	171 (57.8%)	OR: 1.09 (0.82, 1.45)	0.560	139 (16.5%)	OR: 0.16 (0.12, 0.20)	**0.000**
NAS, n (%)	17 (3.1%)	7 (2.4%)	OR: 0.75 (0.30, 1.84)	0.527	26 (3.1%)	OR: 1.03 (0.55, 1.96)	0.917
Fetal distress, n (%)	96 (17.5%)	41 (13.8%)	OR: 0.73 (0.49, 1.09)	0.122	119 (14.1%)	OR: 0.73 (0.54, 0.99)	**0.042**

IQR = interquartile range, n = number, NAS = neonatal abstinence syndrome, sd = standard deviation, 95%CI = 95% confidence interval, coeff = coefficient, OR = odds ratio

^a^ Adjusted for smoking status, parity, maternal age and mental health hospitalisation five years prior to conception

^b^ Data not reported to maintain anonymity

The bold *p*-value indicates a statistically significant result (*p* < 0.05)

The neonates of the continuers and ceasers had comparable levels of admittance to the SCU (OR: 1.09; 95%CI: 0.82, 1.45; *p* = 0.560) and fetal distress (OR: 0.73; 95%CI: 0.49, 1.09; *p* = 0.122) (Table 3). The neonates from the unexposed group were 84% less likely to be admitted to the SCU (OR: 0.16; 95%CI: 0.12, 0.20; *p* < 0.001) and to exhibit signs of fetal distress (OR: 0.73; 95%CI: 0.54, 0.99; *p* = 0.042) than those from the continuers (Table 3).

All other health outcomes of the neonates of the continuers did not significantly differ from those of the ceasers or the unexposed group. This includes prematurity (including very preterm birth) and birth size (birth weight, birth length and head circumference). Apgar scores at one minute and five minutes were not significantly different between the groups. There were also no differences in terms of length of stay in hospital, or NAS diagnosis. Perinatal mortality was uncommon, with less than five cases in each group.

## Discussion

### Main findings

Continued DEX treatment for ADHD during pregnancy did not appear to be associated with negative maternal or neonatal health outcomes when compared to the group that ceased DEX during pregnancy. Thus, women with ADHD may not need to be discouraged from continuing DEX during pregnancy, as we found no association with an increased risk of severe adverse maternal or neonatal health outcomes, although future research is required to support this. However, there does appear to be advantages (e.g. decrease risk for preeclampsia, hypertension, postpartum haemorrhage, admissions to SCU and fetal distress) in ceasing dexamphetamine use prior to pregnancy.

### Strengths and limitations

This was a large study, including all eligible women within WA over a 16-year period. However, it utilised routinely-collected administrative data, of which some information is not collected and thus could not be examined or controlled for. This included information on the exposure (e.g. actual medication use, medication adherence, and timing of medication cessation in relation to outcome assertainment), outcomes of interest (e,g, pregnancy loss prior to 20 weeks), and confounders (e.g. severity of ADHD, and alcohol consumption during pregnancy). Importantly, unmeasured differences between the groups may have also contributed to differences in both maternal and neonatal outcomes. Furthermore, while the study utilised whole-population data, the sample size was not sufficient to robustly examine several rare outcomes including perinatal mortality, hypertension (only for the ceasers), and very early pre-term birth. This small sample size also limited the study with how it was able to match for potential confounders.

It is unclear how generalisable the results from this study would be to other populations, particularly, where the patient population and the use of DEX differs from WA. Differences in treatment regimes, doses, and access to health care may affect outcomes associated with the use of DEX in pregnancy.

### Interpretation

#### Maternal health

Maternal health and characteristics of labour and delivery were typically similar between the three groups. However, women who ceased DEX treatment during pregnancy had a 2.2-fold increased risk of threatened abortion (vaginal bleeding prior to 20 weeks gestation) compared to women who continued DEX treatment. This finding is particularly noteworthy, as it may be associated with the DEX withdrawal process. Although, it could also be explained that women who experienced a threatened abortion were more likely to cease their medication after the event. It was not possible with the existing study data to examine the timing of the threatened abortion in relation to the cessation of DEX in our cohort. Additionally, it is unclear if the risk of threatened abortion was also reflective of an increased risk of spontaneous abortion, which is an important outcome to consider for future research.

A Danish study documented that the risk of miscarriages (loss of pregnancy prior to 20 weeks gestation) was higher among women who continued ADHD medication during pregnancy compared to those who were not exposed to ADHD medication (OR: 2.07; 95% CI: 1.51, 2.84)(Hærvig et al. 2014). However, only MPH, atomoxetine and modafinil were investigated, which could potentially have different mechanisms of how they affect the woman and neonate leading to differing outcomes than that seen with DEX here (Diav-Citrin et al. 2016; Hærvig et al. 2014; Li et al. 2020). Another possibility for the conflicting results is the section of the comparison group without ADHD (Hærvig et al. 2014). This likely resulted in confounding, with these comparison women differing substantially from medicated women with ADHD (Hærvig et al. 2014).

Women in the unexposed group were less likely to develop preeclampsia compared to the continuers, however, there was no difference between the continued and ceased groups for this outcome. A study completed by Camacho et al. ( 2023) found an increased risk of developing preeclampsia in women exposed to stimulant medication compared to those not exposed. This study did not separate exposure to MPH and DEX to investigate if there was a difference between the stimulant medications (Camacho et al. 2023). Another study completed by Cohen et al. ( 2017) also showed similar findings, with women exposed to a psychostimulant having an increased risk of developing preeclampsia compared to unexposed women. A previous study completed by Poulton et al. ( 2018) in Australia found an increased risk for developing preeclampsia if the woman continued ADHD psychostimulant medication throughout pregnancy compared to those who were not treated, however, they were unable to discern if it was the treatment itself that caused this or a direct cause of ADHD. Results from our study, in alignment with previous studies (Camacho et al. 2023; Cohen et al. 2017), indicate that the vasoconstrictive properties of DEX and not ADHD itself may play a role in increasing the risk of developing preeclampsia in the continuers, as the women in the unexposed comparison group were all diagnosed with ADHD but had no increased risk of preeclampsia.

The risk of developing clinically diagnosed hypertension was greater in the continuers compared to the unexposed group. In keeping with this, Newport et al. ( 2016) found that women exposed to psychostimulants during pregnancy were at a greater risk of developing hypertensive disorders during pregnancy than women who did not have any exposure during pregnancy. Hypertension has been associated with psychostimulant medication in nongravid adults, therefore, it is understandable to see it also develop in pregnant women who are taking DEX (Newport et al. 2016). There was also no significant difference between the continuers and ceasers with regard to hypertension. However, these results are based on small numbers and needs to be examined with care.

Continuers had an increased risk for postpartum haemorrhage compared to the unexposed group. However, there was no significant difference between these groups when it came to antepartum haemorrhage. This was an unexpected result, contrasting the results of a previous Australian study, which found no increased risk of postpartum haemorrhage associated with ADHD medication use in pregnancy compared to those who were untreated (Poulton et al. 2018). Further investigation of this outcome is required to determine the potential cause.

#### Neonatal health

The continuation of DEX during pregnancy appeared not to be associated with an increased risk of severe adverse neonatal health outcomes compared to ceasing DEX during pregnancy. The neonates of the continuers had slightly shorter average lengths of gestation compared to neonates from both ceasers and unexposed groups. However, the difference was minimal (0.3–0.4 weeks) and is unlikely to be clinically significant considering there was no difference in preterm birth between groups. This was an unexpected outcome as previous studies showed a risk of preterm birth associated with the continuation of ADHD medication during pregnancy opposed to no treatment (Cohen et al. 2017; Garey et al. 2020; Humphreys et al. 2007; Jiang et al. 2019; Nörby et al. 2017b; Oei et al. 2012; Poulton et al. 2018).

Neonates of the continuers had a greater risk of presenting with fetal distress and being admitted to the SCU compared to neonates from the unexposed group. Although, neonates of the ceasers had a similar risk of these outcomes when compared to continuers. This aligns with a few studies that found ADHD medication exposure *in utero* to be associated with an increased risk for neonatal admittance to the SCU compared to the unexposed group (Jiang et al. 2019; Nörby et al. 2017b; Poulton et al. 2018). This increased risk has previously been explained by DEX crossing the placenta and affecting neonate neurotransmitters, leading to withdrawal symptoms that require additional care (Jiang et al. 2019; Li et al. 2020; Shah and Yates 1978). However, in this study, there was no association between prenatal exposure to DEX with an increased risk of NAS diagnosis when compared to the unexposed group. Fetal distress may contribute to the increased risk of being admitted to the SCU, but fetal distress is a broad term and the exact reasons for the fetus showing distress could not be captured in this study. Further analysis of the reason for SCU admission for these children is required.

## Conclusion

Despite guidelines typically suggesting that were possible patients should cease using DEX during pregnancy, in this study, approximately two-thirds of women continued treatment. Continuing DEX during pregnancy for the treatment of ADHD was not associated with an increased risk of severe adverse neonatal and maternal health outcomes when compared to women ceasing DEX during pregnancy. Although cessation of DEX may be associated with a greater risk of threatened abortion compared to those that continue DEX, indicating a possible association with DEX withdrawal. However, this is something that requires further investigation due to the small sample size, difficulties examining timing, and the inability to examine spontaneous abortion. The use of DEX during pregnancy was associated with an increased risk of preeclampsia, hypertension, postpartum haemorrhage, admittance to SCU and fetal distress, when compared with the non-exposed comparison group, suggesting that where possible the cessation to DEX prior to pregnancy may be advisable. This emphasises the importance of tailoring treatment plans for each woman, as they do have diverse range of personal experiences, expectations, and levels of risk.

## Data Availability

The datasets generated during and/or analysed during the current study are not publicly available due to data custodian concerns surrounding patient confidentiality.

## Code availability

Code is available from the corresponding author on reasonable request.
